# Understanding Barriers and Facilitators to Breast and Cervical Cancer Screening among Muslim Women in New York City: Perspectives from Key Informants

**Published:** 2017-02-23

**Authors:** Nadia Islam, Shilpa Patel, Quanza Brooks-Griffin, Patrice Kemp, Victoria Raveis, Lindsey Riley, Sindhura Gummi, Potrirankamanis Queano Nur, Joseph Ravenell, Helen Cole, Simona Kwon

**Affiliations:** 1 Department of Population Health, NYU School of Medicine, USA; 2 Division of Cancer Prevention & Control, National Center for Chronic Disease Prevention & Health Promotion, Centers for Disease Control & Prevention, USA; 3 Colleges of Dentistry and Nursing, New York University, USA; 4 Lennox Hill Hospital, USA; 5 Kalusugan Coalition, USA

**Keywords:** Muslim, Mammogram, Pap smear, Pap test, Cancer screening

## Abstract

**Background:**

Muslims are one of the fastest growing religious groups in the US. However, little is known about their health disparities, and how their unique cultural, religious, and social beliefs and practices affect health behaviors and outcomes. Studies demonstrate Muslim women may have lower rates of breast and cervical cancer screening compared to the overall population.

**Methods:**

The purpose of this study was to: 1) conduct key-informant interviews with Muslim community leaders in New York City (NYC), to understand contextual factors that impact Muslim women’s beliefs and practices regarding breast and cervical cancer screening; and 2) inform the development and implementation of a research study on breast and cervical cancer screening among Muslims. Twelve key-informant interviews were conducted. The sample included imams, female religious leaders, physicians, community-based organization leaders, and social service representatives. The interview guide assessed: 1) unique healthcare barriers faced by Muslim women; 2) cultural and social considerations in conducting research; 3) potential strategies for increasing screening in this population; and 4) content and venues for culturally tailored programming and messaging.

**Results:**

Key informants noted structure and culture as barriers and religion as a facilitator to breast and cervical cancer screening. Themes regarding the development of targeted health campaigns to increase screening included the importance of educational and in-language materials and messaging, and engaging mosques and religious leaders for dissemination.

**Conclusion:**

Although Muslim women face a number of barriers to screening, religious beliefs and support structures can be leveraged to facilitate screening and enhance the dissemination and promotion of screening.

## Introduction

In 2011, over 220,000 US women were diagnosed with breast cancer and over 40,000 women died, and over 12,000 US women were diagnosed with cervical cancer and over 4,000 women died. There are disparities in cancer burden among different groups cancer morbidity and mortality varies by factors such as socioeconomic status, race, and ethnicity [1–3]. Literature suggests cultural and religious beliefs may impact health-seeking and screening behavior and contribute to health disparities in breast and cervical cancer among racial, ethnic, and religious subgroups [4–6]. Exploring the impact of religious and cultural beliefs on breast and cervical cancer screening within unique religious and ethnic communities are important to cancer control and will contribute to ameliorating health disparities.

One of the fastest growing religions in the United States (US) today is Islam [7]. Because religious affiliation is not systematically tracked in the US, estimates of the size of the Muslim community range anywhere from 1.5 to 9 million [8,9]. There is significant ethnic, social, and cultural diversity within the Muslim community [10]. Sixty-five percent of US Muslims are first-generation immigrants from over 68 nations around the world.[11] Among foreign-born Muslims, the largest groups are from: 1) the Arab region (37%), including Middle Eastern and North African countries; 2) the South Asian region (27%) including Afghanistan, Bangladesh, India, and Pakistan; and 3) the non-Arab regions of Africa (6%) [5,11]. African-Americans make up 35–40% of the US native-born Muslim population,[10,11] Eighteen percent identify themselves as white, non-Hispanic, 10% as Asian, 10% as Hispanic and the rest as mixed race or some other race or ethnicity [12]. Currently, the largest concentration of Muslims in the US resides in New York City (NYC) and the surrounding the tri-state area [13,14].

Previous research has reported that Muslim women in the US, and NYC specifically, face numerous language and healthcare access barriers [15–17], and cultural and religious beliefs impact health attitudes and health-seeking behavior [18]. Societal and cultural norms regarding modesty of dress, how women should interact with males other than their husbands, and beliefs about privacy of the body may restrict Muslim women’s use of healthcare in the US [19–24]. Further, Muslim women may face interpersonal and institutional discrimination on account of gender, ethnicity and/or faith [20]. Studies have documented that wearing hijab (head covering and modest dress) may subject women to discrimination and harassment [17,24] and can affect receipt of social and health services [22].

Although data on breast or cervical cancer morbidity and mortality statistics for Muslims and Muslim subgroups is limited [23], research suggests Muslim women have lower rates of timely breast and cervical cancer screening compared to the overall population [15,22,25–27]. Social, cultural, and religious factors serve as both barriers and facilitators to receiving timely breast and cervical cancer screening in this community. Studies have demonstrated that income and lack of knowledge about screening adversely affect timely cancer screening while factors such as English language proficiency and physician recommendation are facilitators [23,28,29]. In terms of the impact of religion on screening, Underwood and colleagues found that Muslim women reported a lack of participation in breast cancer screening programs because these programs were inconsistent with Islamic beliefs and customs [23]. Similarly, other studies have reported that Muslim women do not receive cancer screening because exposing the relevant body parts may be perceived as a violation of modesty. Such beliefs can result in feelings of anxiety and embarrassment, disenfranchisement from the healthcare system, and low cancer screening rates [18,30,31]. However, research has also suggested that faith can be a facilitator of breast and cervical cancer screening as Islam stresses individual responsibility in health promotion and disease prevention [9].

In this paper we report on the results of key-informant interviews with health care providers, religious leaders, and community advocates that serve the Muslim community in NYC. The interviews were designed to explore the contextual factors that impact Muslim women’s beliefs and practices regarding breast and cervical cancer screening. The investigation also gathered information on community-based strategies that could be used to promote breast and cervical cancer screenings.

## Methods

Data for this analysis comes from the Muslim Americans Reaching for Health and Building Alliances (MARHABA) study, a two year research study supported by the Centers for Disease Control and Prevention’s Prevention Research Center (PRC) Program. The goal of the MARHABA study is to understand the barriers to and facilitators of breast and cervical cancer screening among Muslim women in NYC. The study was guided by a Community Based Participatory Research (CBPR) framework. In CBPR studies, diverse stakeholders with various knowledge and expertise partner to understand community concerns and develop action-oriented solutions to address them [32–34]. The project was co-led by NYU School of Medicine and a coalition consisting of representatives from several social service agencies and leaders in the Muslim community in NYC. The key informant interviews were conducted in the first phase of the project to ensure that the study was guided by community input and voice.

### Sample

Leaders in the Muslim community were purposively sampled to ensure the sample was comprised of a range of representatives. In order to be eligible for the study, individuals had to self-identify as a community leader who worked with the NYC Muslim community for at least five years. Additionally, key informants were selected to represent the various racial and ethnic communities that comprise the NYC Muslim population. Potential participants were identified using a combination of recommendations from MARHABA coalition members and snowball sampling. Using these techniques, twelve key informants were recruited into the study.

### Data Collection

Interviews typically ranged from 30 to 60 minutes. We developed a semi-structured interview guide to gather key contextual information on the Muslim community as well as on the cultural and social factors that impact breast and cervical cancer screening in this population. The interview guide focused on: 1) unique healthcare barriers faced by Muslim women; 2) cultural and social considerations in conducting research in the Muslim population; 3) potential strategies for increasing screening in this population; and 4) content and venues for culturally tailored programming and messaging.

We obtained ethical approval for the study from the Institutional Review Board of NYU School of Medicine in December 2012. Informed consent was not collected as part of the study as no personal health information was collected as part of the interview. The key informant interviews were conducted either in-person or over the phone by the Project Coordinator, between January and February 2012. Participants were given a $40 gift card as compensation for participating in the interview.

### Data Analysis

Transcripts of each interview were created and three members of the research team coded the transcripts. Prior to beginning the actual coding process, the coders independently reviewed the transcripts to establish a general tone. The coders then compiled a comprehensive list of topics present in the transcripts to ensure the creation of an inclusive set of codes. We began the coding analysis by identifying segments of text that related to a unique core code that represented a phenomenon of central interest. For each core code, we developed secondary codes that represented either more specific or restricted aspects of the phenomena. The list of codes was reviewed by research team. The three coders collaboratively drafted definitions for each core and secondary code, and these definitions were also reviewed by research team. The transcripts were reviewed and coded by three people for themes related to barriers and facilitators of breast and cervical cancer screening. Narrative analysis techniques were utilized whereby segments of text relating to themes were identified and core codes and secondary codes were assigned. Relationships between codes within themes were also explored [35]. Discrepancies in coding were resolved within the three coders and the Principal Investigator of the project to reach consensus. To maintain consistency throughout the coding process, we double-coded all of the transcripts.

## Results

### Sample Characteristics

Twelve key informant interviews were conducted. Table 1 presents demographics of the key informant sample. The sample was primarily females and included imams, female religious leaders, physicians, community-based organization leaders, and social service representatives that served a large Muslim population in NYC. (Table 1).

Our narrative analysis of the key informant interviews yielded a rich description of the contextual factors that impact breast and cervical cancer screening in the Muslim community and the potential community-based strategies that could be implemented to promote screening. Two themes emerged related to barriers to breast and cervical screening: (a) structural barriers, and (b) socio-cultural barriers, and several subthemes. The analysis also yielded two themes, with specific subthemes, related to implementing an effective targeted cancer screening campaign in this community: (a) components to include in a targeted health campaign, and (b) dissemination strategies. These various themes and subthemes are listed in Tables 2 and 3, respectively. They are discussed in greater detail below with illustrative quotations drawn from the key informants’ accounts.

### Barriers to breast and cervical cancer screening

The key informants discussed a number of unique barriers faced by Muslim women to obtaining breast and cervical cancer screening. These various barriers fell into two broad categories: structural barriers and socio-cultural barriers (Table 2).

Key informants reported language, insurance, immigration status and limited social networks were specific structural barriers that NYC Muslim women experienced when seeking breast and/or cervical cancer screening. Most commented that a lack of translated information and access to native speaking healthcare providers were significant barriers. They explained that seeking healthcare services would be “*easier*” (KI ID 7) if it was in the women’s own language, but screening information was often “*not translated in a language they understand*,” (KI ID 8). Another common barrier participants mentioned was insurance and cost, explaining that usually Muslim women did not have insurance which meant they were not getting the screenings. “*Insurance-they either do not have it or don’t have enough of it. They also don’t know where to get it either*,” (KI ID 4). Participants also noted that immigration status was often a barrier for many women as they may not seek screenings because of a fear of deportation. “*Some (women) are afraid to come out to get health care because of their immigration status. We need to make it better for them*,” (KI ID 6). Immigration status was also related to having a limited social network to draw upon to get assistance or guidance in obtaining health screenings. “*Muslim women don’t really have big groups of people that they can go to. Some women don’t even have any family here because they are first-generation and no one else is here*” (KI ID 8).

In addition to structural barriers, key informants also discussed a number of socio-cultural barriers that prevented Muslim women from seeking or receiving breast and cervical cancer screening. Nearly all of the participants indicated that many women do not get breast and cervical cancer screening because they do not think it is necessary for their health. Many come from countries where regular screenings do not take place, and thus they were not exposed to the concept of preventive health in their home country. For many NYC Muslim women, screening is a “*novel concept*”, (KI ID 1). Informants reported that women do not go to the doctor unless they are ill, so preventive health care such as screening seems unnecessary.

“*Health is not really seen as preventative. They feel that ‘I am healthy as long as I don’t have to see a doctor.’ That’s why chronic illness is so hard-they don’t understand that this is something that they are going to have to deal with for a long time. That it doesn’t just end. For most, if there is a problem in their health, you just fix it and then you are done with it*” (KID ID 1).

Several of the key informants also reported that fatalism was common among Muslim women. Given this attitude/belief, developing cancer would be viewed as inevitable, and consequently women would not get screened because they cannot avoid their fate. “*They (Muslim women) believe in fate-if something will come, it will come and Allah will help us*” (KI ID 7). Most key informants also noted that socio-cultural gender norms regarding the female role were a barrier. According to many key informants, the female is expected to put the health of their family before their own because… “*Your (Muslim woman’s) own health is still not as important as the health of others,*” (KI ID 12). Because of this, many Muslim women delay or do not seek preventive health care services such as breast and cervical cancer screening: “*There is a cultural expectation for women to put family before themselves. They delay the test like a mammogram or Pap smear because of other obligations. It is not something that the culture enforces but is expected. There is not as much stigma around breast cancer. There is a stigma in women putting themselves first. Other people may look down on them because they are not putting family first*” (KI ID 8).

Because screening requires exposing sensitive areas of the body, Muslim women are embarrassed talking about or engaging in screening tests. As many stated, they are not “*open-minded*” about getting screening (KI ID 4), and “*the whole process of getting a mammogram is embarrassing and women don’t want to do it sometimes*” (KI ID 7). Key informants also noted that women do not want to talk about breast cancer because “*Talking about the breast or the body is shameful and they don’t want to talk about it*” (KI ID 12). Further, although one informant stated that “*there is not as much stigma around breast cancer*” (KI ID 8), most of the key informants did share that there was stigma associated with breast and, in particular, cervical cancer. Specifically, if a woman is diagnosed with breast or cervical cancer, key informants explained that many Muslim women feel that the woman somehow deserved it because she did something “*bad*” (KI ID 1). This may cause rejection from family and/ or friends. “*Apart from the fear of rejection from the partner, there is also the feeling that women are fooling around before or after the marriage. Men always blame women for sexual issues rather than themselves, so women don’t get the screenings*” (KI ID 3). Because of this, many Muslim women will wait to get the screening until it gets to an advanced stage, when treatment may be more complicated [36].

For cervical cancer in particular, key informants reported stigma as a barrier to getting a Pap smear. They noted that Muslim women attribute cervical cancer to bad behaviors, so they do not believe that they need to get screened because they have not engaged in those behaviors. “*Women can sometimes attribute cervical cancer to multiple partners. Their partner is not monogamous, they get HPV, and then cervical cancer so they associate it (cervical cancer) with bad habits. They think, ‘Why should I get screened? I am not doing anything bad,*” (KI ID 1). Key informants also reported gender of healthcare providers was a barrier in breast and cervical cancer screening for Muslim women. Specifically, if Muslim women do not have a female provider, they will not go for care. *“*…*women want to know if a woman or man does the screening. The woman even wants to make sure that there are no men around even when they are at the appointment*” (KI ID 10).

### Strategies to increase breast and cervical cancer screening in the Muslim population

Informants also provided important insight into potential approaches to a tailoring health communication campaign to increase breast and cervical cancer screening among Muslim women (Table 3).

Given the barriers of lack of knowledge and language reported by key informants, most thought these two elements were necessary components in a tailored health campaign. “*Education is the main factor-if we educate them, they will go earlier*” (KI ID 4). A health campaign for this population also had to be translated in different languages. *“Language is the main thing-you need to make sure you tell them about cancer in their own language* …*”* (KI ID 4). In fact, participants thought it was more important to have language-specific over racial- or ethnic-specific materials. *“You can have one program. It is different languages but the same religion”* (KI ID 5).

Beyond language, all participants agreed that the way a health education campaign is presented is integral to its success. In particular, participants had specific ideas on who presents the message and where the message is disseminated. The majority of key informants thought the campaign needed to utilize a “*familiar face*”. “*We should include people from the community. It (the campaign) would not be as effective with new faces because with new faces comes fear. So we should use faces that the women know*” (KI ID 6).

Key informants also reported that certain religious norms could facilitate Muslim women’s receipt of cancer screenings. Islam stresses the importance of personal health, and key informants stated this as a facilitator for screening. “*Health is perceived as positively by the most part. Islam promotes health and taking care of yourself*” (KI ID 10). As such, informants stressed that Islamic religious messages encourage women to get screenings as part of their efforts to stay healthy. Tailored health campaigns for Muslim women could build upon these core religious norms when explaining the importance and health promotion benefits of breast and cervical cancer screening.

Similar to religion being a facilitator to breast and cervical cancer screening, key informants also thought faith-based settings could be an important resource when disseminating information about breast and cervical cancer screening in the Muslim community. All of the participants cited mosques as effective sites for dissemination of a health campaign. “*Mosques would be the best place*” (KI ID 9) as Muslim women often gather there and health campaigns could “*access these clusters and it would be a good way to reach them*” (KI ID 1). Women use their mosque as a “*community center*” (KI ID 3) and the mosque is often “*the only place to go to for socialization and get-togethers*” (KI ID 5) for Muslim women. A few key informants stated that dissemination of a health campaign on breast and cervical cancer screening would be particularly effective during Friday prayer at mosques due to the higher rates of women attending mosques on that day. “*We can do one campaign if we do it within the Masjid [mosque] because it’s the place where people love to be and the place where they love to come*” (KI ID 9).

Participants also thought that the effectiveness of the campaign would be strengthened if certain key stakeholders were engaged, particularly imams, female leaders within mosques and healthcare providers. Specifically, imams should be involved in a health campaign because women see them so frequently at the mosque and “*believe*” in them (KI ID 6). “*The imam could be a great tool because the same person who teaches the way of Islam is now telling the women to get the test*” (KI ID 10). However, key informants noted that imams would have to be educated about breast and cervical cancer screening before they could take part in a health campaign, especially to ensure they would be “*open to the study*,” (KI ID 10). “*If the imams are educated about cancers, they can be used as an asset*…” (KI ID 3).

Almost all key informants thought female leaders in the mosques should be utilized in a health campaign about breast and cervical cancer. Some even thought they would be more effective than imams because women may be hesitant about talking to an imam about breast and cervical cancer. “*For the Muslim general population, lots of women go to the mosque. Not the imam, but female leaders can bring up the topic of breast and cervical cancer. Women would feel free to talk to them and it is more welcoming to talk about the topic because you are speaking in the mosque*” (KI ID 12). Participants also thought healthcare providers should be engaged as they are the “*gatekeepers*” (KI ID3) to screening and “*women get tests because of their medical doctor’s recommendation*” (KI ID 5).

Other venues that key informants thought would be particularly effective for a breast and cervical cancer screening campaign for Muslim women were ethnic media and community based organizations and associations. Although ethnic-specific campaigns were not deemed necessary for the Muslim community, most of the participants did note that ethnic media, including ethnic newspapers, radio, and TV shows, would be an effective vehicle for dissemination. “*In the Bengali community, the newspapers like Tikana and Bangla Patrika would be good. TV shows would be good too. When the women are not going to work or mosque, they are still going to buy newspapers and watch the TV shows*” (KI ID 4). Other community venues such as community based organizations and ethnic associations were also cited as effective locations for health campaign dissemination. “*You can use the community centers that are part of the different country associations, for example the Senegalese Association*” (KI ID 9).

## Discussion

Based on our literature review, this is the first study to report on barriers and facilitators of breast and cervical cancer screening among Muslim women in NYC, where the majority of Muslims in the US reside. Through the use of qualitative and community-engaged approaches, the current study provides important information that will guide development of breast and cervical cancer control initiatives among the Muslim population. Although the key informants were from diverse ethnic and professional backgrounds, they consistently identified the need to address both socio-cultural and structural barriers to breast and cervical cancer screening in the Muslim population. Key informants particularly emphasized the need to address the lack of knowledge about preventive care, language barriers, and gender roles. The importance of leveraging faith-both beliefs and support structures- was emphasized by the key informants as a means of enhancing the dissemination and promotion of breast and cervical cancer screening for Muslim women. Importantly, our study highlights the distinct roles that gender, cultural, and religious norms play in health beliefs and practices. This is an especially important consideration in working with the Muslim population, given its cultural diversity.

There are several limitations to this study. Generalizability may be limited due to the focus on key informants in NYC, which may be a unique setting compared to other areas in the US with high concentrations of Muslims. Second, although a varied group of health care providers, religious leaders and community advocates that serve the Muslim community was interviewed, the study results are limited by the sample size. Nonetheless, given the study’s aim of gaining relative information on barriers and facilitators to breast and cervical cancer screening to inform the development of research and intervention initiatives, the qualitative methodology we utilized in collecting the data and selecting a key informant sample with ethnic and professional diversity are useful in providing information on the Muslim community.

## Conclusions

Findings from our study fill an important gap in the literature about effective means of engaging the Muslim population in health initiatives and understanding the contextual factors that influence Muslim women’s access to preventive cancer screenings. There are only a few studies that have elicited responses from key stakeholders working with this community, particularly related to the development of health campaigns. Following the principles of CBPR, findings from the key informant interviews have been reviewed and interpreted by community partners and will be used to guide the further work with this community. Future phases of this initiative will need to involve in-depth interviews with Muslim women concerning their beliefs and attitudes on breast and cervical cancer screening to further inform the development of a health campaign to increase knowledge and receipt of screening among this population. Given the growth of the Muslim population in the US and evidence which suggests religious beliefs impact breast and cervical cancer screening, CBPR studies that engage diverse stakeholders in the Muslim community such as MARHABA are essential to understanding the barriers and facilitators to breast and cervical cancer screening in this population and to the development of culturally and religiously tailored interventions and messaging to increase screening for these cancers [37].

## Figures and Tables

**Table 1:** Key informant sample demographic information (n=12).

	Frequency
**Sex**	
Female	9
Male	3
**Self-identified Race/Ethnicity**	
African/African American	5
South Asian	7
**Role in Muslim Community**	
Community based organization	4
Female leader in mosque	4
Healthcare provider	2
Imam	1
Social service agency	1

**Table 2:** Barriers to breast and cervical cancer screening among NYC Muslim women (n=12).

	Frequency
**Structural Barriers**	
Language	8
Insurance Status	8
Immigration status	4
Limited social network	2
**Socio-Cultural Barriers**	
Lack of knowledge about preventive care/cancer	11
Gender roles	11
Embarrassment/Stigma	9
Sex of doctors	8
Feelings of fatalism	4

**Table 3:** Necessary components and effective dissemination channels for a targeted health campaign for Muslim women in New York City (n=12).

	Frequency
**Components**	
Educational information on breast and cervical screening	11
Messages and materials in native languages	11
Use of familiar themes and photos	7
**Dissemination channels**	
Mosques	12
Female leaders in mosque	11
Ethnic specific media	10
Imams	8
Healthcare providers	8
Ethnic-specific community-based venues	8

## Abbreviations

CBPR: Community Based Participatory Research
KI: Key Informant
MARHABA: Muslim Americans Reaching for Health and Building Alliances
NYC: New York City
PRC: Prevention Research Center
US: United States
